# Immunohistochemical analysis of protein expression in formalin fixed paraffin embedded human intervertebral disc tissues

**DOI:** 10.1002/jsp2.1098

**Published:** 2020-06-11

**Authors:** Abbie Binch, Joseph Snuggs, Christine L. Le Maitre

**Affiliations:** ^1^ Biomolecular Sciences Research Centre Sheffield Hallam University Sheffield UK

**Keywords:** immunohistochemistry, intervertebral disc, protein localisation, protocol

## Abstract

Immunohistochemistry (IHC) is a useful technique for the localization and semiquantification of protein expression within tissues. Adult human intervertebral disc (IVD) tissues contain a large amount of auto‐fluorescence which often makes immunofluorescence techniques inappropriate on tissue samples but can be applied to isolated cell samples. Thus, IHC remains one of, if not the most common application for protein detection within IVD tissue. Immunostaining localizes antigen expression through specific epitope‐antibody interactions. Within the field of IVD research, IHC is commonly used on fresh frozen and paraffin embedded tissues to elucidate the expression of antigens. Here, we discuss the principles of IHC applied to formalin fixed paraffin embedded IVD tissue and supply optimized protocols for antibodies used within our group to guide research within the IVD field.

## Graphical abstract

Immunohistochemistry of intervertebral disc tissue protocols article.

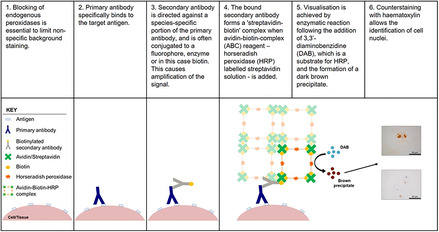

## 1 INTRODUCTION

**FIGURE 1:**
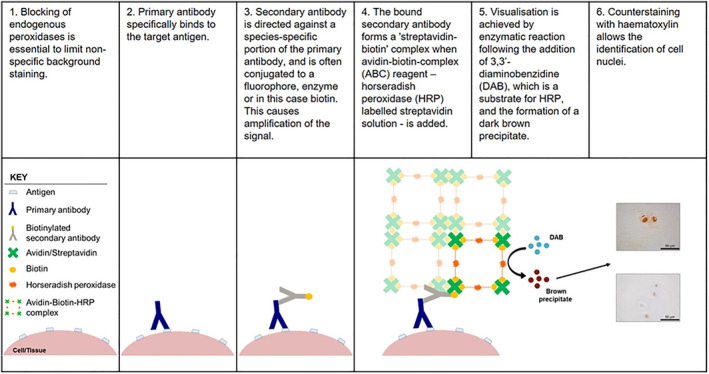
Principle of Immunohistochemistry. Target antigens are detected by the application of specific primary antibodies, followed by the addition of a biotin conjugated secondary antibody which recognizes a specific sequence on the primary antibody. Localisation of bound biotinylated secondary antibody is detected by the addition of streptavidin HRP labeled ABC solution which binds to the biotin on the secondary. Enzymatic color changes occur when DAB reacts with the HRP bound to the secondary antibody, thus resulting in the detection of the target antigen

**FIGURE 2:**
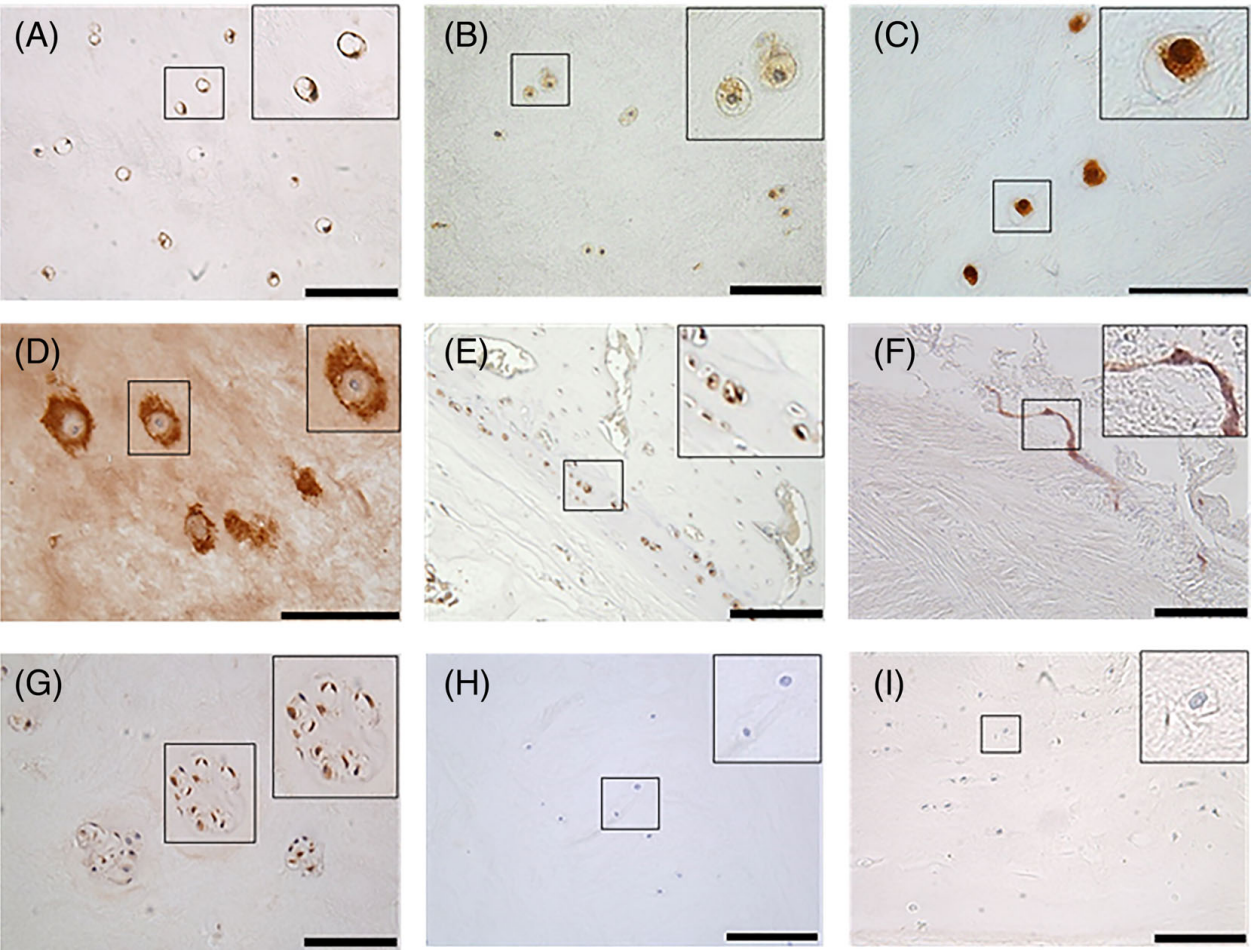
Examples of immunostained formalin fixed paraffin embedded intervertebral disc tissue sections. A, Aquaporin 7 (Abcam ab85907) cell membrane staining, canine nucleus pulposus (NP) tissue. B, Cytoplasmic Indian Hedgehog (Santa Cruz sc‐1196) staining, human NP tissue. C, Nuclear localisation of TonEBP (Abcam ab3446), human NP tissue. D, Pericellular and matrix staining of collagen type II (Abcam ab34712), human NP tissue. E, Staining of aquaporin 6 (Abcam ab191061) indicating cartilaginous endplate (CEP) localisation, canine IVD tissue. F, CD31 (Abcam ab28364) staining within human annulus fibrosus (AF) tissue indicating the presence of endothelial cell infiltration. G, CCL3 (Abcam ab32609) staining within cell clusters, human NP tissue. H, Mouse IgG3, Kappa isotype control (Abcam ab18394), human NP tissue. I, Rabbit IgG isotype control (Abcam ab37415), human NP tissue. A, B, E, F, G, H, I scale bar = 100 μm. C, D scale bar = 50 μm

Immunohistochemistry (IHC) is a widely used technique by which target antigens in tissue can be detected using specific antibodies. Primary antibodies are applied to tissue sections which specifically bind to the target antigen. Application of a secondary antibody directed against a species‐specific portion of the primary antibody is then applied. Secondary antibodies are conjugated, often to a fluorophore, enzyme or biotin, thus allowing the detection and localization of the bound primary antibody. The method of detection used in this protocol utilizes a biotin conjugated secondary antibody which enables an amplification step using the ability of streptavidin to bind to four biotin molecules,^1^ this enables amplification of the signal detected utilizing horseradish peroxidase enzyme (HRP) conjugated to streptavidin‐biotin complex.^1^ This enzyme then converts the substrate, 3,3′‐diaminobenzidine tetrahydrochloride (DAB), into a permanent brown precipitate on the tissue (Figure 1). Counterstaining with hematoxylin allows for the determination of cellular localization and semiquantification of immuno‐positive (Figure 2, brown cellular staining) and immuno‐negative cells (Figure 2, purple/blue stained cell nuclei) (Table 1: IHC protocol). This article aims to provide an overview of the principles of IHC and a clear methodology for performing IHC, for a wide range of antibodies, on formalin fixed paraffin embedded (FFPE) sections of intervertebral disc (IVD) tissue previously optimized by our research group.

**TABLE 1:** IHC Protocol for formalin fixed paraffin embedded IVD tissue all suppliers are provided in Table 2

Step	Action	Timings
1	De‐wax sections in sub‐X	3 × 5 min
2	Rehydrate sections in 100% IMS/ethanol	3 × 5 min
3	Block endogenous peroxidases: Submerge in 100% IMS/ethanol containing 3% (v/v) H_2_O_2_ and 0.06% (v/v) of concentrated HCl	30 min
4	dH20	5 min
5	TBS (20 mM Tris, 150 mM NaCl, pH 7.5) wash (on flat bed shaker)	3 × 5 min
6	Antigen retrieval:	
	None: proceed to step 8	‐
	Enzyme: Preheat 1× TBS buffer containing 0.1% (w/v) CaCl2 dihydrate and 0.01% (w/v) α‐chymotrypsin at 37°C	30 min
	Heat: Preheat buffer containing 0.05M Tris, pH 9.5 to 60°C. Submerge slides, irradiate for 5 min at 40% power in 800 W microwave, leave to stand for 1 min, irradiate at 20% for another 5 min. Leave to cool for 15 min at room temperature.	26 min
7	TBS wash (on flat bed shaker)	3 × 5 min
8	Block nonspecific antibody‐protein interactions	1–2 hours
9	Remove excess blocking solution by tapping slides on tissue paper.	‐
10	Apply primary antibody or appropriate IgG controls	Overnight 4°C
Day 2		
11	TBS wash (on flat bed shaker)	3 × 5 min
12	Apply conjugated secondary antibody (Table 3)	30 min
13	TBS wash (on flat bed shaker)	3 × 5 min
14	Apply ABC elite reagent	30 min
15	TBS wash (on flat bed shaker)	3 × 5 min
16	Apply DAB solution	20 min
17	H_2_O wash (running cold tap water)	5 min
18	Counterstain nuclei with Mayer's hematoxylin	1 min
19	“Blue” sections under running cold tap water	5 min
20	Dehydrate sections in 100% IMS/ethanol	3 × 5 min
21	Clear sections in sub‐X	3 × 5 min
22	Mount sections with coverslips using a mounting medium compatible with sub‐X, for example, Pertex or DPX	‐

## 2 TISSUE PREPARATION

### 2.1 Fixation

IHC can be performed on either frozen or FFPE tissue sections, typically FFPE samples are preferred for analysis of IVD tissue as this enables long term storage of samples and improved morphological maintenance during sectioning of particularly large tissue samples. Following fixation in 10% w/v formalin or 4% (w/v) paraformaldehyde (all reagent suppliers and product codes listed in Table 2). It is essential to ensure appropriate fixation; uneven fixation will result in unreliable IHC staining and lead to difficulties in troubleshooting. Over fixation may lead to brittle tissue sections. During fixation it is important to ensure sufficient volume of fixative to sample with a recommended 50:1 ratio of fixative to tissue sample.^2^ Small fragments of disc tissue obtained from surgery such as discectomy, can be fixed within 48 hours while large whole IVDs should be fixed for a week to ensure complete fixation. For large samples where 50:1 ratio of fixative is not practical, the fixative should be changed every 48 hours, until fixative remains clear.

**TABLE 2:** List of suggested reagents for use with IHC protocol

Reagent	Supplier	Catalogue number
10% neutral buffered formalin	Leica Biosystems	3800600
4% paraformaldehyde	Fisher	15434389
EDTA	Sigma Aldrich	E9884
Sub‐X	Leica Biosystems	3803670
IMS	Fisher	M/4450/17
H_2_O_2_	Sigma Aldrich	H1009
Tris	Fisher	T/3710/60
NaCl	Fisher	S/3120/60
CaCl_2_ dihydrate	Honeywell	223 506
α‐chymotrypsin	Sigma Aldrich	1002472729
Goat serum	Sigma Aldrich	G9203
Rabbit serum	Sigma Aldrich	R9133
Donkey serum	Sigma Aldrich	D9663
BSA	Sigma Aldrich	A2153
Primary and secondary antibodies	See Table 3	See Table 3
VECTASTAIN Elite ABC HRP reagent	Vector Laboratories	PK‐7100
DAB	Sigma Aldrich	D5905
Mayer's hematoxylin	Leica Biosystems	3801582E
Pertex	Leica Biosystems	3808707E

### 2.2 Decalcification

If vertebral bone is included with the IVD sample such as cadaveric discs, a form of decalcification may be required. Decalcification of bone can be achieved using many different reagents that require varying lengths of time to fully decalcify specimens. Neutral 20% (w/v) EDTA (pH 7) requires a longer decalcification time but causes little tissue damage, whereas acidic decalcification acts rapidly but exceeding the end‐point will lead to extensive tissue damage and a loss of IHC staining intensity and thus EDTA decalcification is recommended.^3^ Complete decalcification should be confirmed by X‐ray analysis prior to embedding into paraffin wax. Decalcification time should be optimized according to the size of the tissue sample.

### 2.3 Embedding and sectioning

Following paraffin embedding thin sections (normally 4 μm thick) are prepared on a microtome and floated on a water bath prior to mounting onto positively charged slides (it is important to use thin sections to ensure antibody penetration). When mounting, extra care should be taken to avoid tissue folding. Where folds are generated these areas of tissue should not be included in downstream analysis as trapping of reagents may lead to nonspecific staining. Following mounting onto slides, sections should be dried for a minimum of 1 week to prevent detachment during staining (longer if heat antigen retrieval methods are to be used).

## 3 DEWAXING AND REHYDRATION

Tissue sections embedded in paraffin wax should be deparaffinized in xylene or a xylene substitute such as Sub‐X or Histoclear (Leica) prior to removal of xylene in alcohol (Industrial methylated spirts used here).

## 4 BLOCKING ENDOGENOUS PEROXIDASES

When enzymatic detection methods are utilized, it is essential that endogenous enzymes are removed from tissues to prevent nonspecific staining when the chromogen is added. The enzyme used in this protocol is HRP and thus endogenous peroxidases are exhausted by immersing slides into 3% (v/v) hydrogen peroxide (Sigma, UK) in ethanol (Fisher Scientific, UK) containing 0.06% (v/v) HCl for 30 minutes. Sections are then washed once in deionized water and then twice in Tris‐Buffered Saline (TBS: 20 mM Tris [Fisher Scientific, UK]; 150 mM NaCl [Fisher Scientific, UK]; pH 7.5) which ensures sections are fully rehydrated prior to further steps.

## 5 ANTIGEN RETRIEVAL

As formalin fixation results in crosslinking of proteins this can lead to antigen masking. Furthermore, antigens can be hidden by extracellular matrix components or cellular membranes in the case of intracellular proteins. Therefore in many cases it may be necessary to unmask the antigen by performing an antigen‐retrieval technique.^4^ There are multiple antigen retrieval methods available which can be divided into chemical, enzymatic and heat retrieval methods.^5^, ^6^, ^7^ Antigen retrieval methods are determined for each individual antigen and antibody; no antigen retrieval is also tested during optimization as this can also produce sufficient staining for some antibodies and antigens. Here, we describe the key methods utilized in the protocols for IVD tissue within our laboratory. It is our experience that these methods work reliably for IVD tissues, heat retrieval can be problematic leading to tissue section lifting and should only be used when necessary. It is recommended to leave sections to dry for longer periods if heat retrieval is required, steaming methods can be particular problematic for disc tissue as they often lead to tissue swelling and dissociation from slides.

### 5.1 Heat antigen retrieval

Sections should be placed into a slide rack ensuring that 3 to 4 blank slides are placed at either edge of the rack to reduce the effect of bubbling on tissue sections (edges are affected to a greater extent and this can lead to tissue dissociation if slides with sections are placed in the whole rack). Furthermore, if the rack is not full, extra microscope slides should be added to all empty spaces to allow for even heat distribution across all sections. The slide rack is then immersed in 400 mL antigen retrieval buffer (0.05 M Tris HCl, pH 9.5, preheated to 60°C), and irradiated for 5 minutes at 40% power in a Sanyo 800 W microwave oven (adjust time and power according to Watt output of microwave). Sections are left to stand at room temperature for 1 minute, before being irradiated for a further 5 minutes at 20% power. Sections are left to stand for 15 minutes within the hot buffer to cool.

### 5.2 Enzyme antigen retrieval

Sections (in a slide rack) are placed in TBS containing 1% (w/v) α‐chymotrypsin and 0.1% CaCl_2_ (preheated to 37°C) and incubated for 30 minutes at 37°C.

## 6 ANTIGEN DETECTION

Following antigen retrieval, sections are washed three times in TBS and then placed into humidified slide boxes. Nonspecific protein interactions are blocked and secondary antibody host interactions are neutralized by the application of 200 μL 1% (w/v) BSA in 75% v/v TBS and 25% (v/v) normal serum (matched to the species which the secondary antibody was raised in) and incubated for 1 to 2 hours at room temperature. Optimum antibody concentrations are predetermined by optimizations whereby a range of concentrations and antigen retrieval methods are tested for optimal staining (a list of preoptimized antibodies is documented in Table 3). Primary antibodies are diluted in 1% (w/v) BSA in TBS and sections incubated overnight at 4°C to enable binding of antibody to antigens. Parallel sections should be incubated in IgG controls (Figure 2B) to ensure primary antibodies are not binding nonspecifically. It is important that tissue samples do not dry out at any point, if drying occurs this can lead to nonspecific staining which often appears as high intensity staining around the edge of the tissue.

**TABLE 3:** Conditions and antibody dilutions optimized using the immunohistochemistry method outlined in this article

Reference	Target antibody	Clonality	Optimal dilution	Antigen retrieval	Secondary antibody	Optimal dilution
*AF markers*						
[11]^a^	Collagen typae I (ab90395)	Mouse monoclonal	1:200	Enzyme	Rabbit anti mouse (ab6727)	1:500
[12]^b^	Collagen type I (ab34710)	Rabbit polyclonal	1:200	Enzyme	Goat anti rabbit (ab6720)	1:500
*Bone markers*						
[12]^b^	Alkaline Phosphatase (ab108337)	Rabbit monoclonal	1:200	Heat	Goat anti rabbit (ab6720)	1:500
[12]^b^	Collagen X (ab49945)	Rabbit polyclonal	1:400	Enzyme	Goat anti rabbit (ab6720)	1:500
[12]^b^	Osteocalcin (ab13420)	Mouse monoclonal	1:400	Enzyme	Rabbit anti mouse (ab6727)	1:500
[12]^b^	Osteopontin (ab69498)	Mouse monoclonal	1:200	None	Rabbit anti mouse (ab6727)	1:500
[12]^b^	Runx2 (ab76956)	Mouse Monoclonal	1:200	None	Rabbit anti mouse (ab6727)	1:500
*Cell cycle and apoptosis*						
[11, 13]^a^	Caspase 3 (ab13847)	Rabbit polyclonal	1:400	None	Goat anti rabbit (ab6720)	1:500
Unpublished	P16INK4a (ab108349)	Rabbit monoclonal	1:100	None	Goat anti rabbit (ab6720)	1:400
*Cell signaling*						
[14]	c‐jun (ab32385)	Rabbit polyclonal	1:400	Heat	Goat anti rabbit (ab6720)	1:300
[15]^c^	IHH (Santa Cruz, sc‐1196)	Goat polyclonal	4ug/mL	Enzyme	Donkey anti goat (ab6884)	1:400
[9]	NFkB (ab31481)	Rabbit polyclonal	1:100	Heat	Goat anti rabbit (ab6720)	1:300
[11]	NOTCH2 (ab8926)	Rabbit polyclonal	1:200	Heat	Goat anti rabbit (ab6720)	1:400
[9]	P38 MAPK (ab4822)	Rabbit polyclonal	1:800	Heat	Goat anti rabbit (ab6720)	1:300
[15]^c^	PTCH (Santa Cruz, sc‐6149)	Goat polyclonal	1:50	Heat	Donkey anti goat (ab6884)	1:300
[15]^c^	PTHrP (Santa Cruz, sc‐9680)	Goat polyclonal	1:25	Heat	Donkey anti goat (ab6884)	1:300
[15]^c^	PTHR1 (Santa Cruz, sc‐12 777)	Goat polyclonal	1:20	Heat	Donkey anti goat (ab6884)	1:300
[15]^c^	SMO (Santa Cruz, sc‐6366)	Goat polyclonal	1:25	Heat	Donkey anti goat (ab6884)	1:300
*Cytokines and chemokines*						
[16]	CCL2 (ab9669)	Rabbit polyclonal	1:500	Heat	Goat anti rabbit (ab6720)	1:500
[17]	CCL3 (ab32609)	Rabbit polyclonal	1:4000	Heat	Goat anti rabbit (ab6720)	1:500
[17]	CCL4 (ab9675)	Rabbit polyclonal	1:2000	Heat	Goat anti rabbit (ab6720)	1:500
[16]	CCL7 (ab104866)	Rabbit polyclonal	1:10000	Heat	Goat anti rabbit (ab6720)	1:500
[18]	CCR1(ab89055)	Mouse monoclonal	1:1000	Heat	Rabbit anti mouse (ab6727)	1:400
[16]	CXCL8 (ab7747)	Rabbit polyclonal	1:100	Heat	Goat anti rabbit (ab6720)	1:500
[18]	CXCR1 (ab60254)	Mouse monoclonal	1:2000	Heat	Rabbit anti mouse (ab6727)	1:400
[18]	CXCR2 (ab24963)	Mouse monoclonal	1:500	Heat	Rabbit anti mouse (ab6727)	1:400
[11]^a^	IL‐1β (ab9722)	Rabbit polyclonal	1:100	Heat	Goat anti rabbit (ab6720)	1:500
[11]^a^	IL‐1RI (ab100278)	Rabbit polyclonal	1:100	Enzyme	Goat anti rabbit (ab6720)	1:500
[16]	IL‐16 (ab9563)	Rabbit polyclonal	1:750	Heat	Goat anti rabbit (ab6720)	1:500
*Immune markers*						
[18]	CD4 (ab51312)	Mouse monoclonal	1:500	Heat	Rabbit anti mouse (ab6727)	1:400
Unpublished	CD11b (ab62817)	Goat polyclonal	1:800	None	Donkey anti goat (ab6884)	1:500
[12]^b^	CD68 (ab955)	Mouse monoclonal	1:200	Enzyme	Rabbit anti mouse (ab6727)	1:500
*Matrix remodeling*						
[11]^a^	ADAMTS4 (ab185722)	Rabbit polyclonal	1:200	None	Goat anti rabbit (ab6720)	1:500
[11]^a^	MMP3 (ab53015)	Rabbit polyclonal	1:400	Enzyme	Goat anti rabbit (ab6720)	1:500
[11]^a^	MMP13 (ab39012)	Rabbit polyclonal	1:200	Heat	Goat anti rabbit (ab6720)	1:500
*Neural and vascular*						
[19]	NF200 (ab82259)	Mouse monoclonal	1:400	Heat	Rabbit anti mouse (ab6727)	1:500
[19]	PGP9.5 (ab8189)	Mouse monoclonal	1:200	Heat	Rabbit anti mouse (ab6727)	1:500
[19]	CD31 (ab28364)	Rabbit polyclonal	1:400	Enzyme	Goat anti rabbit (ab6720)	1:500
[20]	Sema3C (ab135842)	Rabbit polyclonal	1:200	None	Goat anti rabbit (ab6720)	1:500
[20]	Sema3D (antibodies online—ABIN1386639)	Rabbit polyclonal	1:200	Enzyme	Goat anti rabbit (ab6720)	1:500
[20]	NRP‐1 (ab81321)	Rabbit monoclonal	1:200	Heat	Goat anti rabbit (ab6720)	1:500
[20]	NRP‐2 (ab185710)	Rabbit polyclonal	1:200	Enzyme	Goat anti rabbit (ab6720)	1:500
[20]	Plexin A1 (ab32960)	Rabbit polyclonal	1:200	Enzyme	Goat anti rabbit (ab6720)	1:500
[21]	Substance P (ab10353)	Mouse monoclonal	1:500	None	Donkey anti mouse (Alexa Fluor 488)	1:200
Unpublished	NGF (ab52918)	Rabbit monoclonal	1:100	Enzyme	Goat anti rabbit (ab6720)	1:500
Unpublished	VEGF (ab52917)	Rabbit monoclonal	1:100	Enzyme	Goat anti rabbit (ab6720)	1:500
*NP markers*						
[11, 13]^a^	Aggrecan (ab3778)	Mouse monoclonal	1:100	Heat	Rabbit anti mouse (ab6727)	1:500
[13]^a^	Chondroitin sulphate (ab11570)	Mouse monoclonal	1:400	Enzyme	Rabbit anti mouse (ab6727)	1:500
[11]^a^	Collagen type II (ab34712)	Rabbit polyclonal	1:200	Enzyme	Goat anti rabbit (ab6720)	1:500
[13]^a^	Collagen type II (ab3092)	Mouse monoclonal	1:200	Enzyme	Rabbit anti mouse (ab6727)	1:500
[10, 21]^a^	FOXF1 (ab23194)	Rabbit polyclonal	1:100	Heat	Goat anti rabbit (ab6720)	1:500
[11]^a^	HIF1α (ab16066)	Mouse monoclonal	1:100	None	Rabbit anti mouse (ab6727)	1:500
[22]	KRT‐19 (ab7754)	Mouse monoclonal	1:400	None	Rabbit anti mouse (ab6727)	1:500
[22]	LAM‐5 (ab78286)	Mouse monoclonal	1:800	Enzyme	Rabbit anti mouse (ab6727)	1:500
[10, 21]^a^	PAX1 (ab203065)	Rabbit polyclonal	1:400	Enzyme	Goat anti rabbit (ab6720)	1:500
*Osmotic regulation*						
[23]^c^	AQP0 (ab134695)	Rabbit polyclonal	1:200	Enzyme	Goat anti rabbit (ab6720)	1:500
[23, 24]^c^	AQP1 (ab15080)	Rabbit polyclonal	1:400	None	Goat anti rabbit (ab6720)	1:500
[23]^c^	AQP2 (ab85876)	Rabbit polyclonal	1:400	None	Goat anti rabbit (ab6720)	1:500
[23]^c^	AQP3 (ab125219)	Rabbit polyclonal	1:1600	Enzyme	Goat anti rabbit (ab6720)	1:500
[23]^c^	AQP4 (ab9512)	Mouse monoclonal	1:200	Enzyme	Rabbit anti mouse (ab7074)	1:500
[23]^c^[24]	AQP5 (ab92320)	Rabbit polyclonal	1:100	Heat	Goat anti rabbit (ab6720)	1:500
[23]^c^	AQP6 (ab191061)	Rabbit polyclonal	1:200	Enzyme	Goat anti rabbit (ab6720)	1:500
[23]^c^	AQP7 (ab85907)	Rabbit polyclonal	1:100	Enzyme	Goat anti rabbit (ab6720)	1:500
[23]^c^	AQP9 (ab85910)	Rabbit polyclonal	1:400	Enzyme	Goat anti rabbit (ab6720)	1:500
[25]	TonEBP (ab3446)	Rabbit polyclonal	1:100	Heat	Goat anti rabbit (ab97049)	1:500
*Ca* ^*2+*^ *channels*						
Unpublished	TRPV1 (ab3487)	Rabbit polyclonal	1:100	Enzyme	Goat anti rabbit (ab97049)	1:500
Unpublished	TRPV4 (ab94868)	Rabbit polyclonal	1:200	Enzyme	Goat anti rabbit (ab97049)	1:500
*Lactate transport*						
[26]	MCT1 (Santa Cruz, sc‐50 324)	Rabbit polyclonal	1:20	Heat	Goat anti rabbit (ab97049)	1:500
[26]	MCT4 (Santa Cruz, sc‐50 329)	Rabbit polyclonal	1:10	Heat	Goat anti rabbit (ab97049)	1:500
[26]	CD147 (Santa Cruz, sc‐9754)	Goat polyclonal	1:10	Heat	Donkey anti goat (ab6884)	1:500

*Note*: All antibodies optimized for use on formalin fixed paraffin embedded (FFPE) human intervertebral disc (IVD) tissue.

^a^ Publications referenced report these antibodies in optimized for use on human mesenchymal stem cells embedded within hydrogel.

^b^ Optimized for use on murine bone tissue.

^c^ Also optimized for use on canine IVD tissue. All antibodies supplied by Abcam unless stated otherwise.

### 6.1 Detection of bound primary antibody

All sections are washed three times in TBS on an orbital shaker, before biotinylated secondary antibody is applied for 30 minutes at room temperature. Antibody dilutions are performed in 1% (w/v) BSA in TBS.

### 6.2 Visualization of bound secondary antibody

Following secondary antibody application, sections are washed three times in TBS before 2 to 3 drops of ABC Elite Reagent (Vector Laboratories, UK) is applied for 30 minutes at room temperature. The addition of avidin‐biotin‐complex (ABC) reagent (HRP labeled streptavidin solution) allows the formation of a “streptavidin‐biotin” complex with the secondary antibody. Sections are washed three times prior to application of 200 μL per section of 0.08% (v/v) hydrogen peroxide in 0.65 mg/mL DAB in TBS for 20 minutes. The enzymatic reaction between DAB and HRP forms a permanent dark brown precipitate allowing visualization of bound antibodies. Sections are washed in dH_2_O for 5 minutes prior to immersion in Mayer's Hematoxylin (Leica, UK) for 1 minute and blued under running tap water for 5 minutes.

## 7 MOUNTING OF SECTIONS

Sections are dehydrated, cleared and mounted in Pertex (Leica, UK).

## 8 EVALUATION OF IHC

In addition to utilizing IHC to determine localisation of proteins of interest, semiquantitative analysis can be performed. For each primary antibody, 200 cells within each region of interest (eg, nucleus pulposus/annulus fibrosis/cartilaginous endplate) are counted. It is also possible to analyze cell clusters and single cells separately depending on your hypothesis. To ensure avoidance of selection bias for areas of staining, the first field of view of the tissue of interest should be the start point of analysis. Analysis of that tissue area should then be performed in a raster fashion to ensure no selection or double counting. Cells are counted as immunopositive (brown) or immunonegative (Purple nuclei counter stain only) (Figure 2) and the percentage of immunopositive cells can be calculated and plotted. As percentage data is utilized for quantification, data is not normally distributed and thus means and standard errors should not be used. Graphical representation should show all data points or utilize a box and whisker plot or other graph suitable for nonparametric data.

For the quantification of extracellular proteins such as extracellular matrix markers cellular immunopositive staining is still useful as this indicates current cellular production of these proteins (as long as the antigen detects the intracellular form). This can be complemented by percentage area of immunopositivity which can be quantified using ImageJ software. The use of image intensity for IHC analysis should be avoided for ABC/DAB methodology as the signal is an amplified signal and intensity cannot be directly related to quantity of protein.

### 8.1 Automated IHC analysis

While there are a number of automatic image analysis systems which enable automatic IHC quantification for tissues, which is particularly used in the cancer fields.^8^ These systems can be problematic for disc tissues. Disc tissues contain few cells (~4 × 10^6^ cells/cm^3^) in the NP,^9^, ^10^ which means either large numbers of images must be captured and analyzed, or full slide analysis is required. For the majority of laboratories who do not have facilities for rapid reliable full slide image capture it is often more time and cost effective to manually quantify immunopositivity down the microscope. Furthermore, it is important to accurately identify tissue type of interest (eg, NP, AF, and CEP) during analysis and this can be just as time consuming to “label” tissue areas within image capturing software.

## 9 STATISTICAL ANALYSIS

As data is not normally distributed (percentage data) data should be analyzed using nonparametric testing dependant on the groups and hypothesis being tested. For example, where investigating immunopositivity between grades of histological degeneration if more than two groups are analyzed a Kruskal‐Wallis test with appropriate posthoc analysis can be performed when a significant difference is seen between study groups. Linear regression analysis can be used to observe correlations between for instance, percentage immunopositivity and grade of degeneration, and correlation between target molecules or age.

## 10 CONCLUSIONS

IHC is a reliable technique which can provide key insights into the expression and localization of proteins within cells and tissues. While semiquantifiable it can provide key indications of changes in cellular expression of proteins during disc development, disease and regenerative strategies. IHC can also be an essential tool to help characterize cells and garner an understanding of their behavior in their natural state. However, it is essential to ensure samples are processed appropriately, thin flat sections are prepared, and antibodies selected and optimized to ensure complete antigen retrieval and avoiding nonspecific background staining, supported with appropriate controls.

## CONFLICT OF INTEREST

There are no conflict of interest to declare.

## AUTHOR CONTRIBUTIONS

All authors contributed to the optimization of IHC described within the protocol and all authors contributed to manuscript preparation. Christine L. Le Maitre secured funding. All authors read and approved the final manuscript.
